# *In vivo* Pooled Screening: A Scalable Tool to Study the Complexity of Aging and Age-Related Disease

**DOI:** 10.3389/fragi.2021.714926

**Published:** 2021-08-31

**Authors:** Martin Borch Jensen, Adam Marblestone

**Affiliations:** ^1^ Gordian Biotechnology, San Francisco, CA, United States; ^2^ Astera Institute, San Francisco, CA, United States; ^3^ Federation of American Scientists, Washington D.C., CA, United States

**Keywords:** *in vivo*, pooled screening, direct *in vivo* screening, aging models, animal models, gene therapy, single cell sequencing, barcoding

## Abstract

Biological aging, and the diseases of aging, occur in a complex *in vivo* environment, driven by multiple interacting processes. A convergence of recently developed technologies has enabled *in vivo* pooled screening: direct administration of a library of different perturbations to a living animal, with a subsequent readout that distinguishes the identity of each perturbation and its effect on individual cells within the animal. Such screens hold promise for efficiently applying functional genomics to aging processes in the full richness of the *in vivo* setting. In this review, we describe the technologies behind *in vivo* pooled screening, including a range of options for delivery, perturbation and readout methods, and outline their potential application to aging and age-related disease. We then suggest how *in vivo* pooled screening, together with emerging innovations in each of its technological underpinnings, could be extended to shed light on key open questions in aging biology, including the mechanisms and limits of epigenetic reprogramming and identifying cellular mediators of systemic signals in aging.

## Introduction

**FIGURE 1:**
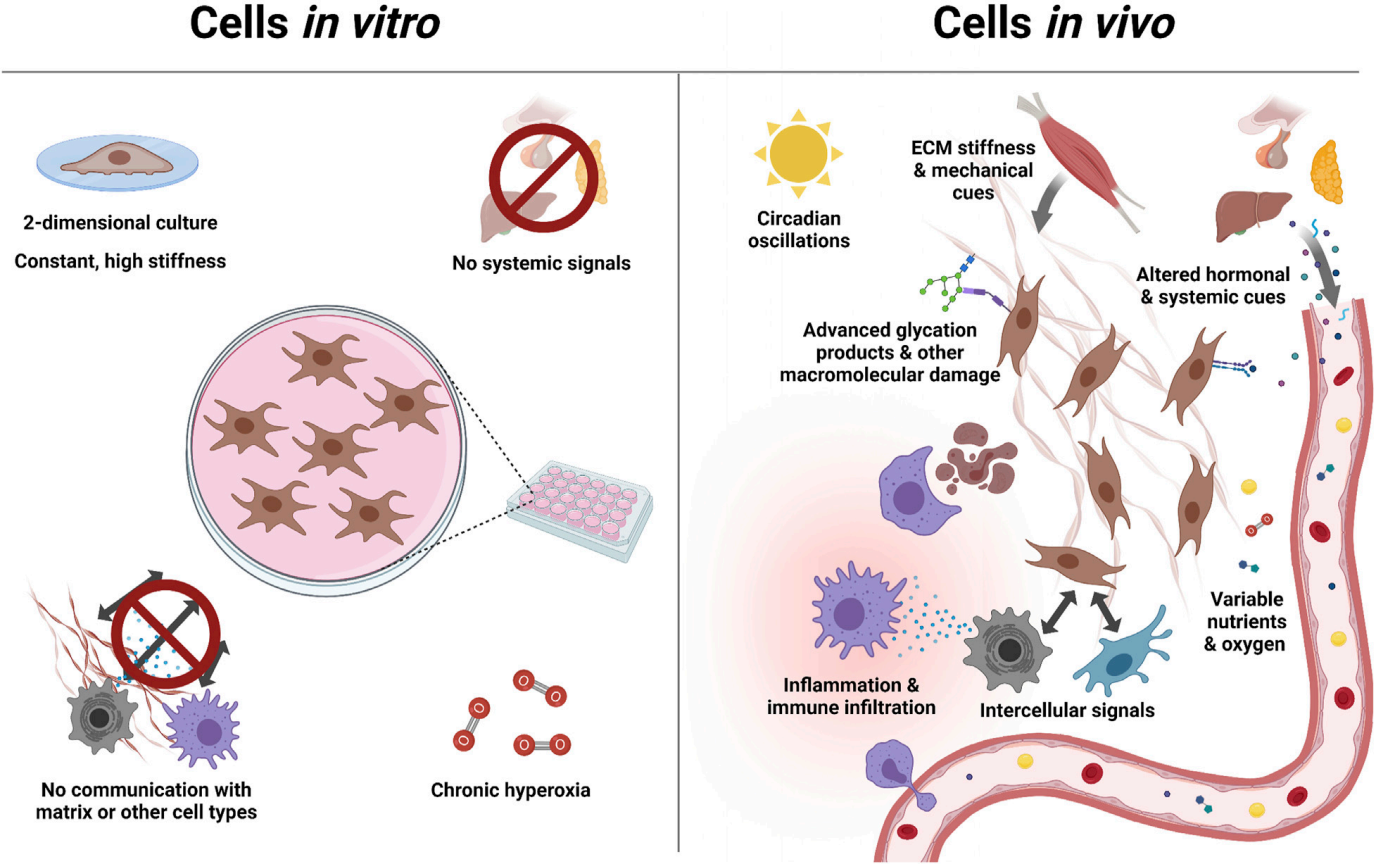
Complexity of aging is overlooked by *in vitro* models: Aging *in vivo* exposes cells to diverse stimuli, which drive and regulate the aging process. Most of these stimuli, including signals from other cells and organs, are absent *in vitro*. This can in some cases be addressed with specialized growth conditions (e.g. in hydrogels), or by co-culturing 2 cell types. But no current system addresses all of these factors, let alone factors we have yet to discover. This motivates the study of aging in its natural, *in vivo*, environment.

The complexity of aging biology creates challenges for the study of age-related disease. Aging takes place over long timescales, in a complex systemic and local tissue environment. This means that the study of cells in isolation likely overlooks the *in vivo* context (Figure 1). For example, signaling between immune and parenchymal cells drive aspects of tissue aging (Buechler, Fu, and Turley 2021; Yousefzadeh et al., 2021), the mechanical stiffness of the local tissue niche impacts the aging of central nervous system progenitor cells (Segel et al., 2019) and development of fibrosis (Wells 2013). Such factors can lead to very different outcomes when studying disease *in vivo* versus *in vitro* (Miller et al., 2017). Meanwhile, complex secretory and immune phenotypes (Covarrubias et al., 2020), or even the activity of specific neurons in the brain (Zullo et al., 2019), can alter cellular metabolism at distal sites (Camacho-Pereira et al., 2016). In addition, different organs, tissues and cell types within those tissues are found to age differently (Rando and Wyss-Coray 2021), and the causal factors of aging are likely multifarious (López-Otín et al., 2013).

While we have seen an explosion of multi-omics analysis in recent years, allowing us to generate large amounts of high dimensional data (Tabula Muris Consortium 2020), distinguishing correlation from causation is a major challenge. Any given metric that changes with age or in an age-related disease could represent a causal driver, could be a compensatory response, or could be an ancillary effect. Ultimately, discriminating these scenarios requires the ability to apply causal perturbations, as a means of hypothesis testing, in the *in vivo* context.

Ideally, to cope with this complexity, many targets could be compared in separate cellular experiments occurring in an intact, living animal, i.e., *in vivo*. Operating directly in the *in vivo* setting means that complex aging biology can be probed directly, without the need to first understand and then translate it to an *in vitro* model. Of course, *in vivo* screening has been a core feature of aging research for decades. Model systems like *C. elegans* have been a mainstay of this approach since they enable many perturbations to be applied, and thus causal hypotheses to be tested, in parallel by a single lab at a reasonable cost (Lucanic et al., 2018). Extending these approaches to larger, mammalian models, however, entails a much greater operational footprint, unless a means can be found to test many hypotheses in the same animal.

In this review, we describe how the emerging paradigm of ***in vivo*** pooled screening can contribute to overcoming these challenges, with a focus on technical considerations that will enable researchers in the aging field to apply these methods in their work. *In vivo* pooled screening builds on two co-occurring revolutions: in multiplexed single-cell analysis, and in multiplexed *in vivo* gene-specific perturbation. In turn, these technologies have only become possible in the past decade due to advances in microfluidics, molecular and cellular barcoding, nucleic acid programmable genetic and epigenetic modulators made possible by CRISPR technologies, as well as multiplexed gene synthesis and the increased availability of gene delivery vectors. We will focus on the two primary challenges to pooled screening *in vivo*: delivering perturbations in a targeted and resolvable manner, and readouts that measure effects on multiple biological processes in individual cells, while giving examples of past and potential applications to understand the biology of aging and age-related disease.

## From Plates to Organs—Moving Screens *in vivo*

**FIGURE 2:**
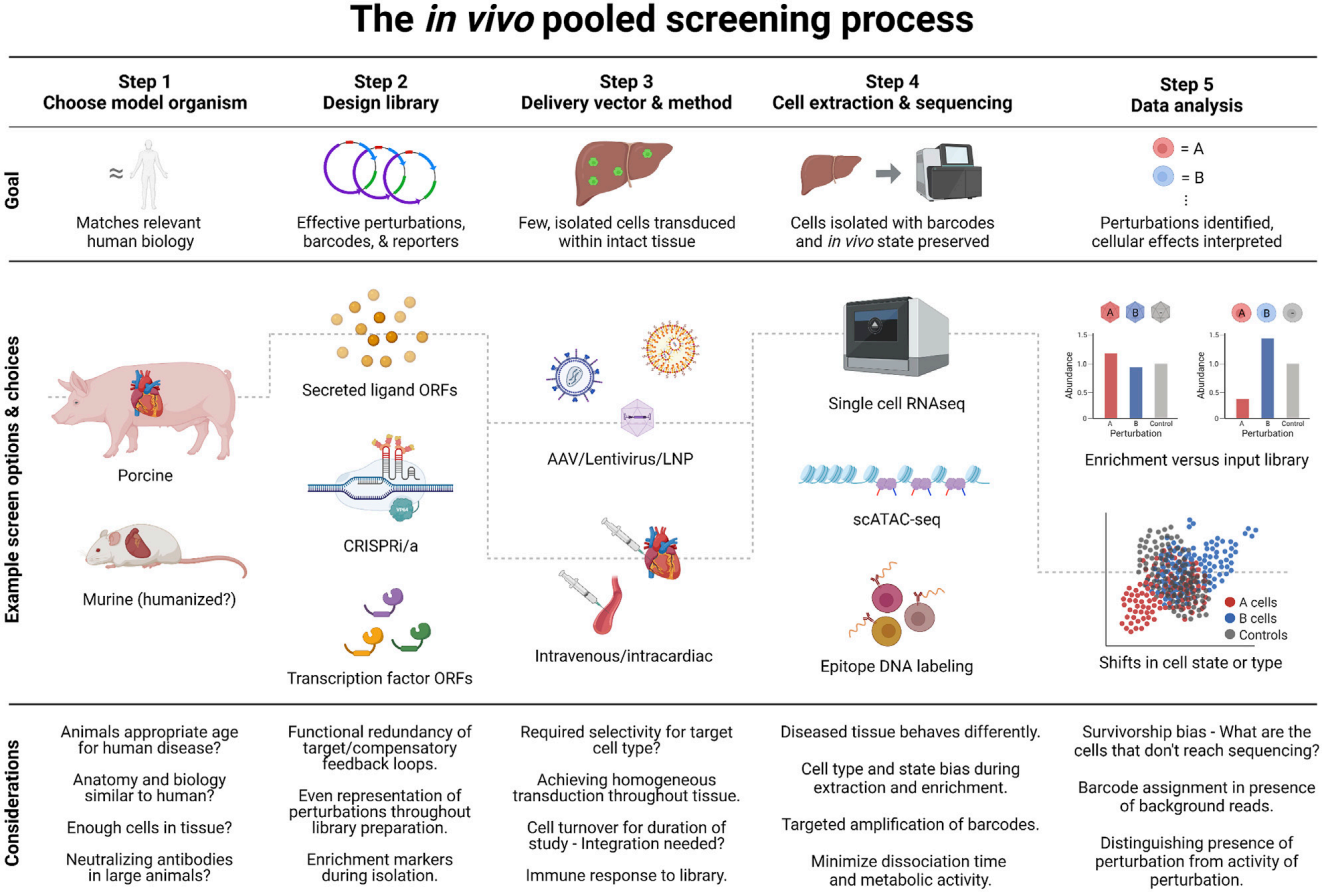
The process of an *in vivo* pooled screen. *In vivo* pooled screens require careful choices at five key steps of the process. To illustrate this in practice, we include the decision-process for a hypothetical screen setup inspired by a published *in vitro* screen (Theodoris et al., 2020): The screen looks for treatments for aortic valve disease caused by *Notch1* loss of function. Since mechanical forces affect the disease, an ideal model would have heart size and structure similar to humans, e.g. a *Notch1* loss of function pig model. To find potential therapies, the screen could focus on secreted ligands, which could be developed as biologics. Suitable barcodes and a fluorescent reporter would also be included in perturbation constructs. Cardiomyocytes are non-replicating and can be transduced well by adeno-associated virus, but no serotype exists that targets these cells without transducing e.g. the liver. Thus intracardiac injection would best limit the perturbations to cardiomyocytes. Previous work had identified a gene network characteristic of disease, so measuring the transcriptomic state of perturbed cells would be the ideal readout. For cardiomyocytes, single-nucleus sequencing is likely to yield better quality data than whole-cell sequencing. This means that the library should be constructed with a nuclear-localized reporter. Additional considerations are noted in the figure, and discussed in the text.

The essence of an *in vivo* pooled screen is to administer a pooled library of different perturbations to a single, living model organism, with a screen readout that can both distinguish the effects of each intervention on the cells that the intervention ended up in, and identify of each intervention. Successful outcomes, meaning accurate and interpretable data, depends on methodological choices at each step of the process shown in Figure 2. This hypothetical example walks through how a published *in vitro* screen for ways to treat age-related calcific aortic valve disease, with *in vivo* validation (Theodoris et al., 2020), could have been designed as a direct *in vivo* screen. The rest of this section provides detailed discussion of the challenges and considerations at each step of this process.

### Identifying Perturbations

A quintessential feature of pooled *in vivo* screening is that the perturbed cells are connected to the larger organ and systemic environment, rather than being isolated in individual plastic wells. This implies imperfect control over which cells end up receiving a specific perturbation, a challenge that is generally addressed by making the perturbations identifiable post hoc. This is most commonly done by coupling expression of the perturbation to a nucleotide “barcode” that can be identified in sequencing-based readouts. Nucleotide barcodes can take the form of DNA or RNA transcripts expressed from DNA, depending on the screen’s readout. They can also encode distinct protein products that are then used as barcodes (Wroblewska et al., 2018). For example, in CRISPR based screening the Protospacer Adjacent Motif, a targeting sequence for Cas9, can uniquely identify each guide RNA expressed in a cell. Identifying barcodes can also be embedded in untranslated regions of RNA polymerase II transcripts if protein-coding libraries are screened, or simply included in integrating segments of DNA. Appropriate choice of barcoding Technology can make or break *in vivo* screening experiments, and will be discussed further in the section on screen readouts.

Although DNA-barcoded libraries of small molecules are commonly used for *in vitro* pooled screens, we are not aware of any reports of *in vivo* use (Goodnow, Dumelin, and Keefe 2017). Likewise, antibodies and other proteins can be tagged with DNA barcodes, but we are not aware of any studies injecting barcoded proteins into living organisms. In both cases, it is likely that the stability and immunogenicity of attached DNA presents a technical challenge that remains to be solved. Further, the *in vivo* half-life of the perturbation molecules themselves would limit their use to screens with a very early readout. One could speculate that replacing DNA barcodes with protein/peptide tags, or simply with variant amino acid sequences, could help enable *in vivo* pooled screening of biologics. And for some purposes, such as optimizing *in vivo* stability, a short half-life could be an advantage. Nevertheless, the remainder of this review will focus on nucleotide based perturbations delivered in gene and cell therapy vectors.

### Factors in Screen Design

Indeed, nucleic acid-based perturbations are far from restrictive: they are able to modulate, in either direction, the activity of almost any gene in the genome, and further to express non-coding RNAs, specific clinical mutations, or even protein variants not observed in nature. The range of targets thus greatly exceeds the 10–20% of the genome that is considered druggable with small molecules and biologics (Finan et al., 2017). Decreased target activity can be accomplished by expressing RNAi constructs, CRISPR editing or epigenetic inactivation (CRISPRi), or by expressing inhibitors or dominant negative versions of the target. Increased target expression/activity can be achieved either by introducing coding sequences for the target (including constitutively active versions), activating endogenous promoters using CRISPRa, or by reducing expression of inhibitors. Additionally, protein variants with altered function, for example receptor variants (Roth et al., 2020), can be explored in their natural environment with direct *in vivo* screening. Guidelines for perturbation libraries used in pooled screening have been covered elsewhere [e.g. (A. Schuster et al., 2019; Hanna and Doench 2020)], and we therefore focus on considerations that apply specifically to screening *in vivo*.

First among these is the immune system. Studying cells that are exposed to the full suite of immune cells is one big advantage of *in vivo* screening, by bringing into play the real physiological responses to inflammation, senescence, and other elements of age-related disease. But both delivery vectors and perturbation payloads can trigger a response from the immune system, hindering delivery of perturbations and/or altering the environment and results of the study. One source of immunogenicity is the introduction of payloads from other species, and especially from microbial species. For example, introduction of (bacterial) Cas9 in CRISPR screens can activate the immune system of mouse models (Chew et al., 2016; Dubrot et al., 2021). Gene transfer and gene editing can also create new antigens, even when introducing human proteins. If the host immune system has been trained on a mutated version, or total absence of that protein, the wild type variant can be recognized as foreign and expressing cells cleared (Fields et al., 2001). Some species and mouse strains are more sensitive to foreign proteins, which may play into the choice of model for screening. It is likewise important to consider the inflammatory state of the tissue to host the screen; if already inflamed, the probability of immune responses to perturbations increases, as does the risk that introduction of foreign elements exacerbates disease state.

Perturbations also interact with *in vivo* biology in ways unrelated to the immune system, wherever their biological features interface with cellular processes. For example, shRNA expression can overload the endogenous microRNA-processing machinery, causing toxicity at high levels but likely affecting microRNA expression even at lower levels (Grimm et al., 2006, 2010). At sufficient levels even overexpression of GFP can cause cellular toxicity (Klein et al., 2006), possibly by overloading protein folding in the endoplasmic reticulum. Promoters used to express perturbations may also behave differently *in vivo* than *in vitro*: since the unnatural environment of cell culture broadly affects gene programs, expression even in primary cells *in vitro* does not ensure expression in the same cell type *in vivo* (Figure 1). Furthemore, promoters may be epigenetically silenced, especially when they are derived from pathogens (Brooks et al., 2004). Each of these considerations will apply differently to different cell types, and are hard to predict without empirical validation. Proper planning of *in vivo* pooled screens thus includes both considerations about the cellular targets one wishes to study (e.g. gain vs. loss of function, signaling redundancies, isoforms), but also how the organism will respond to each element of the perturbation library.

### Delivering Perturbations *in vivo*

Barcodes alleviate the need to perfectly control perturbation delivery but most *in vivo* pooled screens still have target parameters for delivery. These include the multiplicity of infection (for consistency we’ll refer to MOI even for non-viral vectors), i.e. whether each cell receives single perturbations or a combination, whether perturbations are restricted to a certain tissue or cell type, the fraction and absolute number of cells that are perturbed, and the time frame during which perturbations are active. Choice of delivery vector is a major factor in achieving these parameters. We provide an overview of the most popular vectors and their relative advantages in Table 1, and refer the reader to other excellent reviews for additional details (Dunbar et al., 2018; Yin et al., 2014; D.; Wang, Tai, and Gao 2019). In this section, we use examples of pooled *in vivo* screening to highlight considerations for successful delivery.

**TABLE 1:** Delivery modalities for *in vivo* pooled screening

	AAV	Lentivirus	Adenovirus	Lipid nanoparticle (with mRNA/siRNA)	siRNA/antisense oligos
Targetable tissues and cell types	Many (liver, muscle, brain, eye, lung, heart, and more)	Many	Many	Mainly hepatocytes, vasculature reported	Mainly liver and kidney, neurons with direct injection
Inter- and intra-tissue spread	Medium-high	Low	Low	Medium	High
Duration of treatment possible	Stable episomal expression in non-dividing cells for months+	Stable integration in dividing and non-dividing	Stable episomal expression in non-dividing cells for months+	Days to weeks, unless gene editing modalities delivered	Days to weeks
Optimal payload size	4–4.5 kb	7–8 kb	8–30 kb	Any	<100 bp
Payload vector construction	Moderate	Moderate	Hard	Easy	Easy but expensive
Immunogenicity	Low	Medium	High	Low	Low-High

A key challenge for *in vivo* delivery is to achieve selective exposure of delivery vectors to target cells, when the circulatory system provides access to the rest of the body. When possible, replacing intravenous delivery with direct tissue administration helps, but most vectors will still reach other tissues (Rubin et al., 2019; D. J.; Schuster et al., 2014). The simplest strategy to maximize on-target delivery is to expose cells to vector *ex vivo*, and then transplant the modified cells into animals. The number of cells thus modified is typically small, and often the experimental strategy includes expansion of the modified cells either *ex vivo* or after transplantation. Preserving the perturbations during expansion requires integration into the genome, and lentivirus is therefore the most commonly used delivery vector. Single MOI can be achieved by combining a low virus-to-cell ratio with a selection step to eliminate uninfected cells. Common selection methods include fluorescence activated cell sorting or exposure to cytotoxic agents, with the fluorophore and/or resistance gene delivered alongside the perturbation. Transplant-based screens were first performed in the cancer field (Zender et al., 2008; Bric et al., 2009; Meacham et al., 2009; Gargiulo et al., 2014; Chen et al., 2015; Galeev et al., 2016; Grüner et al., 2016), where such experiments are common. An important feature of transplant-based screening is the possibility of using primary or cultured human cells to study the effect of perturbations on human biology. However, such xenografts require immunocompromised host organisms, in turn decreasing the relevance conferred by screening *in vivo*. Transplant-based screens are also used to study the hematopoietic system, both its cancers and normal functions (Adams et al., 2012; Cellot et al., 2013). *In vivo* screens of the immune system hold great promise for studying how both cellular aging and the aged systemic milieu contribute to loss of immune function and discrimination (Miller 1996).

*In vivo* screening can be applied to study aging biology more generally by delivering perturbations directly to animal models. The liver was the first and most common mammalian organ to host such screens (Wuestefeld et al., 2013), for two reasons: First, delivery to hepatocytes is extremely effective. Vectors with large cargo size, and even naked DNA, delivered intravenously readily transduce the liver. Second, because hepatocytes are powerfully regenerative even in adults, improved regeneration can be used as a readout where the signal from positive hits is automatically amplified. No published study has focused on liver aging specifically, but mediators of fibrotic injury have been identified (Zhu et al., 2019).

Moving beyond the liver increases the delivery challenge. Some *in vivo* screens have achieved efficient delivery using lentivirus administered during development (Shah et al., 2015; Jin et al., 2020), but using this approach to study aging would be very slow. Similarly, some screens have combined mice engineered to express Cas9 with guide delivery via AAV (Chow et al., 2017) or lentivirus (Wertz et al., 2020; Keys and Knouse 2021). Cas9 effector-expressing mice are much more versatile than genetically engineered models, because a single mouse line can be combined with different gRNA libraries to perform a variety of screens (Lima and Maddalo 2021). But some features of aging and age-related disease are more accurately modeled in non-mouse models (Chu, Szczodry, and Bruno 2010; Getz and Reardon 2012; Calado and Dumitriu 2013; Basil and Morrisey 2020; Hoffman et al., 2018; Chiou et al., 2020). Thus, for the study of aging and age-related diseases, *in vivo* pooled screens will be most powerful when identifiable perturbations can be delivered in their entirety in a single vector, and thus applied to any animal model. This has recently been accomplished using lentivirus-delivered shRNA in Huntington’s models (Wertz et al., 2020), and AAV-delivered overexpression constructs (Ruozi et al., 2015; Jensen et al., 2020).

The principles of delivering perturbations *in vivo* naturally build upon principles for pooled screening in general: Constructing and packaging the library of perturbations should take care to preserve diversity, the number of cells sampled should be high enough to avoid stochastic differences in how many of each perturbation is included, and dose should be optimized to achieve the desired number of perturbations per cell.

Additional principles apply specifically *in vivo*: Unlike in cell culture, *in vivo* delivery means that different cell types will be exposed to the vector. These cell types may have differences in transducibility, response to perturbations, turnover and proliferation rates, and more. Choice of vector is essential for optimizing delivery to the cells one wants to screen in, and cell-type specific promoters may be required as well. Cellular coverage needs to be considered with diversity of cell states in mind. Avoiding interactions between cells receiving different interventions requires a low rate of transduction, such that non-transduced cells surround every transduced cell. Similarly, simply getting vector to the desired cells (ideally in an evenly distributed manner) can be a challenge. For example, many vectors cannot cross the blood-brain barrier, whereas almost all vectors tend to preferentially transduce the liver. The size of the target tissue may constrain the number of cells available for screening in each animal, although larger animal models can address this. For some administrations, wider diffusion of small vectors such as AAV will be preferred, while other studies will want to constrain delivery to a smaller region (Lerchner et al., 2014). In dividing cell types, integrating vectors are required, and particularly large or complex payloads may require vectors with larger packaging capacity. Otherwise, AAV is often preferred because it is less immunogenic than other viruses and can be used in Biosafety Level 1 environments, although neutralizing antibodies can be present in non-mouse models (Hurlbut et al., 2010).

Finally, the physiological decline that happens with age does create additional challenges. Loss of cells, as in neurodegenerative diseases and osteoarthritis, can make it difficult to recover enough cells to read out a screen. In general, aged and diseased cells are also more difficult to recover by dissociation. In fibrotic diseases, altered and excessive collagen deposition can restrict access to some cells, affecting which delivery vectors can be successfully used. Gene therapy vectors rely on receptors to enter cells, e.g. Ldlr for LNPs (Akinc et al., 2010) and glycan receptors for AAV (Huang et al., 2014), and the expression level of these receptors can change with age and disease. Unfortunately, systematic studies of gene delivery to old organisms have not been reported, so optimization will likely be required before screening. This can be guided by atlases of age-related changes, which already exist for mice (Tabula Muris Consortium 2020). Although inconvenient, the challenges to delivery in aged organisms are part of what makes these models more relevant for human age-related disease.

## Interpreting Biology Through Perturbations

Utilizing the scalability of pooled screening depends on having phenotypic readouts that associate perturbation barcodes with the outcome of perturbation. Most *in vivo* pooled screens thus far have looked at enrichment or depletion of perturbation barcodes after genome integration/editing, representing gain/loss of cellular proliferation (Zender et al., 2008) and/or survival (Wertz et al., 2020). Of these, enrichment is more popular because gain of signal is easier to measure than loss of signal. Enrichment can be measured at a single time point, or after multiple rounds of directed evolution (Ruozi et al., 2015). Enrichment readouts are cost-effective because signal is inherently boosted by the phenotype screened for, and results can thus be robust even when a subset of perturbed cells are sampled.

Nevertheless, there are many sources of noise and artifacts for such screens [covered in detail elsewhere, e.g. (A. Schuster et al., 2019)], and signal amplification is often useful or necessary. *In vitro*, it is common to kill non-transduced cells with e.g. puromycin, but this strategy clearly does not work *in vivo*. Instead, a fluorophore or other sorting tag can be used to select transduced cells. This has the additional advantage of selecting for successful gene expression, where DNA rather than RNA barcodes are used, but note that this selection process will introduce false positives and negatives, and sacrifices some transduced cells through inefficiencies. Typically, PCR amplification of perturbation barcodes prior to sequencing is used to improve depth of sampling. Here, it is essential to use methods that eliminate differences from differing PCR efficiency between barcodes. A standard option is to tag each molecule with a unique molecular identifier (UMI) in the first round of amplification, which then distinguishes the parent molecule after additional cycles (Kinde et al., 2011). It is likewise essential to normalize apparent enrichment to representation of each perturbation in the input vector. Since representation changes at each step of generating delivery vectors (e.g. bacterial expansion and vector packaging), it’s rarely appropriate to normalize to the initial plasmid mix. In principle normalization could be relative to earlier time points. But since assays are typically destructive this relies on comparisons between different animals with physiological variability that is likely to affect readouts and bias the normalization.

In the context of aging, one could imagine using enrichment screens to identify protective factors in degenerative disease, or introducing reporters for e.g. senescent cell states alongside perturbations that induce or prevent that state. But many aspects of aging biology cannot be studied in terms of cellular abundance, and the variety of biological processes involved calls for multidimensional readouts**.** Single-cell RNA sequencing (scRNAseq) can provide this additional layer of interpretability for the effects of perturbations, similar to multivariate imaging screens (Liberali et al., 2015) but in a more realistic *in vivo* setting. Indeed, bulk transcriptomics has already been used not only to identify transcriptomic signatures of aging, but also to identify small molecule interventions that revert transcriptomes to a “younger” state and extend organismal lifespan (Baumgart et al., 2016; Janssens et al., 2019). Single-cell sequencing not only enables *in vivo* pooled screening using such transcriptomic readouts of aging, but also is able to distinguish age-related changes that occur heterogeneously in different cell types (M. J. Zhang et al., 2021; Kimmel et al., 2019) and even in cells of a single type (Enge et al., 2017; Martinez-Jimenez et al., 2017; Kaufmann et al., 2021).

**FIGURE 3:**
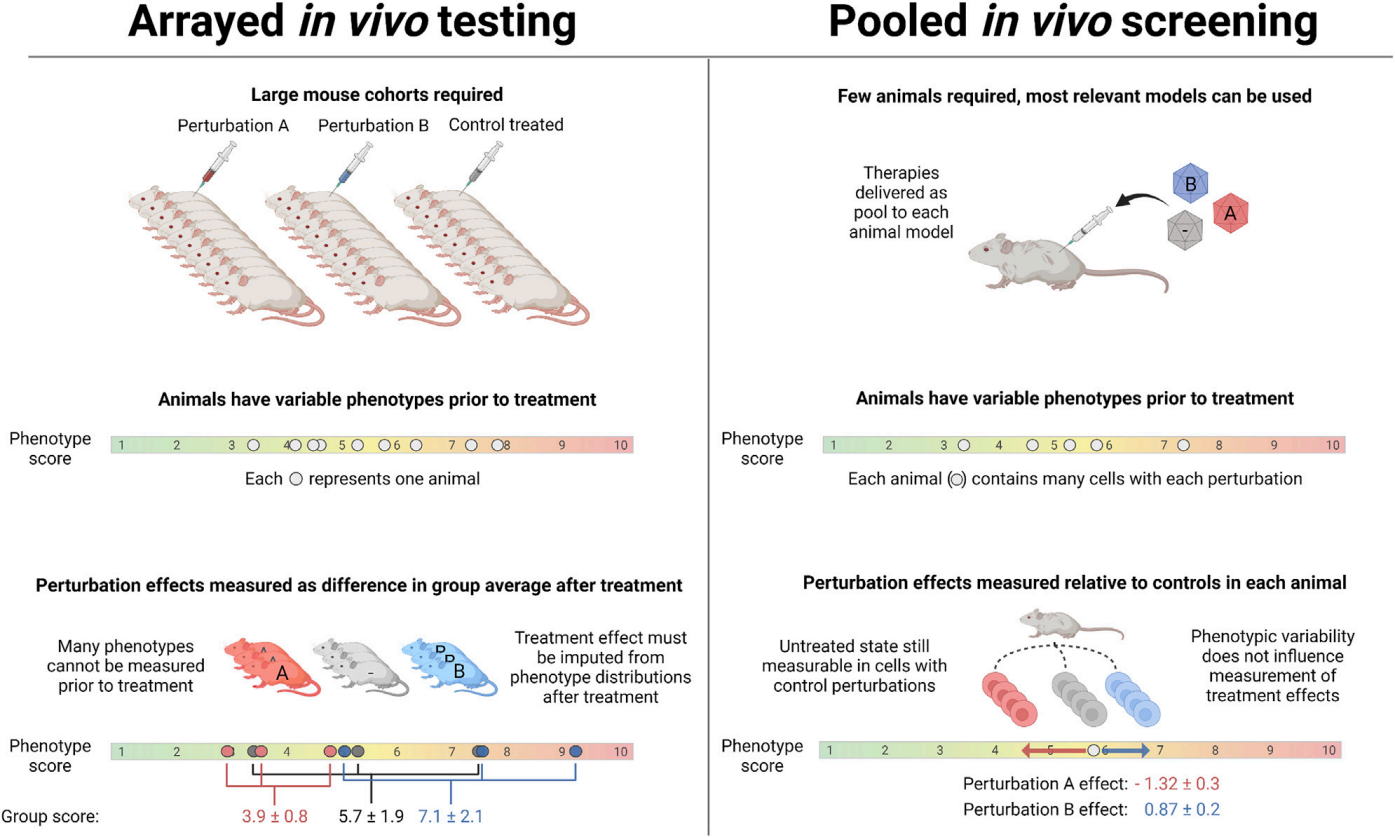
Key differences between pooled and traditional *in vivo* testing: In non-pooled *in vivo* experiments, animal-to-animal variability in phenotypes of interest and in response to perturbation necessitates using large cohorts. With *in vivo* pooled screening, the effect of perturbations is measured relative to unperturbed cells in the same animal, side-stepping inter-animal phenotypic variability. Because large cohorts are not required, the most relevant species and/or animals with spontaneous age-related disease can be used in place of commonly available models.

Perturbation screens with single-cell transcriptomic readouts were originally reported in lentivirus-based *in vitro* screens (Adamson et al., 2016; Dixit et al., 2016; Jaitin et al., 2016; Datlinger et al., 2017), but have recently been applied to *in vivo* systems (Jensen et al., 2020; Jin et al., 2020). In addition to revealing heterogeneous responses of different cell states to a given perturbation, *in vivo* pooled screens allow transcriptomic effects to be evaluated relative to controls within the same animal. Although it is still important to consider environmental factors that affect physiology, such as housing temperature (Seeley and MacDougald 2021) and group size, circadian rhythms, and diet, perturbation effect vectors within outlier animals can still be interpreted. This also greatly facilitates analysis for animal models that manifest a range of disease severities, including naturally occurring rather than experimentally induced disease models (Figure 3).

But, for *in vivo* screens in particular, it’s important to ask whether the sampled cells are representative of the biology one intended to study. Both cell type and state affects viability after dissociation, so the cells that end up sequenced cannot be assumed to be a random sampling of the *in vivo* perturbed population (Meyer and Schumacher, 2021). When integrating vectors are used, cell division during the course of the experiment produces progeny cells that share barcodes and perturbations but may be quite different in terms of aging processes. Similarly, barcode molecules may be detected in immune cells that have consumed the originally transduced cells. And depending on how many cells in a tissue are perturbed, the overall state of the tissue may be shifted, such that the default cell state is no longer representative of the biology one wishes to model. Thus, *in vivo* pooled screens should always be designed with attention to how the host tissue will respond to both delivery, expression, and isolation of perturbations.

Another challenge with droplet-based scRNAseq is that not every RNA molecule in each cell is captured (X. Zhang et al., 2019), including the barcode molecules required for analysis of perturbation effects. Methods that improve expression and/or capture of barcode molecules improve the number of cells where the perturbation can be identified (Replogle et al., 2020). But more sensitive barcode detection can lead to the inverse problem: multiple barcodes detected in droplets, due to RNA from lysed cells or other contaminants. Different methods have been used to distinguish such droplets from “true multi-perturbations”, including UMI cutoffs and Bayesian modeling, but as yet there is no consensus on the best approach.

A related issue is confirming the efficacy of perturbations, e.g. whether a knockout or knockdown has occurred in a cell containing barcodes for a specific perturbation. As a first step, it’s essential to avoid technical artifacts (e.g. PCR template switching) during perturbation library production that lead to inaccurate pairing of barcodes and perturbations (Adamson et al., 2018). But even correctly identified perturbations may be inactive in some cells. Methods used to quantify genome editing (Clement et al., 2020) are difficult to apply to single-cell data, and variation in gene expression between cells makes loss of expression difficult to measure with confidence. One approach is to have barcodes contained in the RNA transcript of the perturbation itself, although this places some constraints on both elements and does not strictly measure activity. Recently, tools have been developed to detect perturbation based on effects on the broader transcriptome (L. Yang et al., 2020a; Duan et al., 2019; Papalexi et al., 2021). This approach can be combined with redundant perturbations for each target, such that response to perturbation of a given target can be distinguished from (in) activity of a specific perturbation.

Using -omic readouts such as scRNAseq in combination with a large number of perturbation generates a wealth of data, which describes comprehensive cell states and regulatory networks responsive to perturbation. This type of data has been used to generate machine learning models predicting the effects of drugs (Lotfollahi, Wolf, and Theis 2019; Theodoris et al., 2020; Burkhardt et al., 2021) and attempt to identify effective therapeutics. *In vivo* pooled screening takes this approach to the next level, by directly measuring the efficacy of perturbations in the physiological environment where they would be needed. As with all computational approaches, machine learning models are powerful only when the data used to train them is appropriate for the attempted predictions. With the right data, machine learning approaches are a promising tool for understanding the interplay of processes that drive age-related decline.

## Applications to Aging Biology

*In vivo* pooled screening as a technology is still in its infancy, and all the more so when applied to the biology of aging. Transcriptomic changes have been used to identify aging therapies using *in vivo* screens in individual model organisms like *C. elegans* (Janssens et al., 2019) and *N. furzeri* (Baumgart et al., 2016; Janssens et al., 2019), and this methodology can be extended to large mammalian species using pooled screens. As described in prior sections, this is already possible and starting to happen using current technologies (Jensen et al., 2020), and ongoing technology trends promise to overcome many current limitations for screen readouts. Table 2 summarizes these emerging prospects. Ultimately, *in vivo* pooled screening approaches could be applied broadly across aging research to interrogate causality in a highly multiplexed fashion, in naturally aged mammalian models.

**TABLE 2:** Emerging readout technologies applicable to pooled screening and selected aging biology applications.

	Targeted single-cell RNA sequencing	Multi-modal single-cell analysis	*In situ* analysis	Molecular recording and lineage tracing
Understanding cell-type-specific, cellular-level mediators of systemic aging perturbations	✓	✓		
Quantitative profiling of the effects of perturbations on expression of genes that change with age in particular cell types and organs	✓			
Understanding post-transcriptional aging pathways		✓	✓	
Developing safe *in vivo* cellular reprogramming methods		✓		✓
Establishing relationships between intracellular and extracellular hallmarks of aging		✓	✓	
Efficiently mapping the functional genomics of aging in long-lived or non-model species	✓	✓		
Studying causal relationships between aging processes		✓	✓	✓

Although pooled screening approaches only multiplex cell-autonomous (also known as “cell intrinsic”) perturbations, and cannot measure organismal lifespan, they could also be used to understand the mechanisms of systemic perturbations that do affect aging and/or lifespan. For example, the use of system-wide perturbations like the application of bloodborne factors or caloric restriction could be combined with pooled CRISPR inactivation screening to determine genetic effectors of such systemic perturbations on particular cell types. This could be done, for example, by comparing the single-cell transcriptome of calorically restricted and ad libitum-fed animals, as has been done in bulk (Hong et al., 2010), and then using single-cell transcriptomic readouts to identify perturbations that eliminate the response to caloric restriction. Such approaches could reveal not only cell-type specific or context dependent regulation, but also common pathways mediating effects of such perturbations across cell types, furthering our understanding of their basic mechanisms of intercellular aging. Previous studies have combined single-cell transcriptomics with bespoke single-cell measurements like optical detection of plasma protein uptake by cells within the brain (A. C. Yang et al., 2020b). While this study suggested potential genetic regulators of plasma protein uptake, the approach was only correlative; *in vivo* pooled screening allows an extension to probe causality, i.e., which genes, in which cell types, modulate plasma protein uptake by CNS cells. Aging research has recently begun to identify many proteins in blood plasma that change with age (Tanaka et al., 2018, 2020; Lehallier et al., 2019; Orwoll et al., 2020), or that modulate aging (Castellano et al., 2017; Vinel et al., 2018), and the possible secretion sources of these proteins from various organs are beginning to be identified based on transcriptomic expression profiling. However, the causal, functional roles of these proteins in the aging process are poorly understood. A pooled screening approach could knock down specific cell surface receptors for these plasma factors and look for reversion of transcriptomic signatures induced by parabiosis (Ferenbach et al., 2016), to determine mediators of parabiosis factors in a massively multiplexed fashion.

While conventional assays physically dissociate a tissue before biochemical analysis, *in situ* assays operate by imaging directly in an intact, often chemically fixed specimen, maintaining access to the three-dimensional spatial context of each measurement. *In situ* readout of pooled screening inside intact tissues could have key advantages, beyond just restoring cells to their spatial contexts. For example, some *in-situ* readouts are able to detect subtler properties of cells, such as the subcellular localization of specific proteins (Reicher et al., 2020) or nucleic acids (Alon et al., 2021) within specific organelles or compartments, or morphological features relevant to aging, like cell membrane shape or nuclear membrane integrity (Faulkner et al., 2021). Such techniques could be used to identify, for example, cytoplasmic DNAs that have been linked to inflammatory processes in aging (Simon et al., 2019). While *in-situ* readout of pooled screening has so far been applied only in isolated cells (Feldman et al., 2019), *in situ* sequencing and multiplexed immunohistochemistry methods are already applied to intact tissues (X. Wang et al., 2018; Alon et al., 2021). *In situ* readout approaches could also be especially powerful for understanding the effects of cell intrinsic processes on the surrounding local environment of that cell (Yousefzadeh et al., 2021). For example, a pooled screening approach with an *in-situ* readout could interrogate the effects of genes on the extracellular matrix composition or accumulation of extracellular aggregates in the neighborhood of a given cell or cell type, or the role of locally secreted factors (C. Wang et al., 2021). When applied to senescent cells, such approaches could probe genes that modulate local effects of the senescence associated secretory phenotype (SASP). Other examples would include the effects of gene perturbations on the local infiltration of immune cells, or the accumulation of lipid droplets, tau tangles, amyloid fibrils or synapse loss characteristic of neurodegeneration. Ultimately, *in situ* readout should allow any assay accessible on fixed tissue *via* an optical microscope to be used as an output for pooled screening.

The application of *in vivo* pooled screening to aging biology would benefit from extension to proteomic readouts as well. Single-cell transcriptomics can now be combined with single-cell surface affinity proteomics, and extensions to affinity proteomics of intracellular proteins are on the horizon (Mimitou et al., 2020; Rivello et al., 2020; Swanson et al., 2021; Stoeckius et al., 2017; Chung et al., 2021). At the simplest level, this could be applied to verify the direct effects at the protein level of the perturbations used in pooled screening, i.e., whether a knockdown of a receptor actually eliminates that receptor from the cell surface, in the example above. But more broadly, much of aging biology likely occurs at the protein level. Long-lived proteins, like nuclear pore complexes, are important in aging (Toyama et al., 2013), and mapping their abundance, and ultimately their subcellular distribution with multiplexed *in-situ* approaches (Goltsev et al., 2018), could provide key phenotypic readouts not accessible to transcriptomics. A pooled screening approach could search for the factors that most impact the states of such long-lived proteins, distinguishing, for example, roles of genes involved in reactive oxygen species (ROS) production, chaperones, or autophagy.

Moreover, key aging regulators like FOXO or mTOR are heavily regulated by protein-protein signaling networks which depend on post-translational modifications (Smith and Shanley 2010; Y.-X. Lu et al., 2021), with key biology likely invisible at the transcriptomic level. In this context, protein based, rather than RNA based, barcoding of pooled screening perturbations should allow the use of immunohistochemistry, rather than RNA sequencing, as a readout of pooled screening. This would open up integration with multiplexed immunohistochemical stains (Wroblewska et al., 2018; Goltsev et al., 2018), or possibly with emerging genome-wide single-cell proteomic approaches (Specht et al., 2021).

Epigenetic clocks measuring DNA methylation provide a putative readout of the biological age of a tissue (Haghani et al., 2021), and such methylation measurements and clocks have recently been extended to the single-cell level (Trapp et al., 2021; Linker et al., 2019), which could provide a direct readout of biological age in pooled screens for aging modulators. Organ-specific and cell-type specific transcriptomic aging clocks are also a possibility, to invoke some integrated measure of cellular age as a readout (Meyer and Schumacher 2021). The sensitivity of single-cell transcriptomics to small variations in gene expression, or to low abundance transcripts, is considerably lower than that of bulk transcriptomics, but targeted sequencing approaches like HyPR-Seq can achieve much greater sensitivity for a defined, yet still diverse subset of genes, such as aging clock gene arrays (Marshall et al., 2020). More complex clock-like signatures computed from comparisons across species or from responses to known aging drugs (Tyshkovskiy et al., 2019) could also be used as readouts for pooled screening. Methylation measurements can be combined with single cell RNA sequencing in a manner that should allow pooled screening (Linker et al., 2019) to identify factors driving the response of epigenetic clocks to aging interventions and explore causality for this metric of biological age.

Relatedly, future application of *in vivo* pooled screening could have special relevance to the biology of *in vivo* cellular reprogramming (Y. Lu et al., 2020; Ocampo et al., 2016), which has recently come to prominence in aging research due to its apparent reversal of epigenetic age and several other aging hallmarks. The current set of transcription factors used for *in vitro* reprogramming may not be optimal *in vivo,* given the risk of teratoma formation following complete reprogramming (Abad et al., 2013). Indeed, there is some evidence that dedifferentiation and rejuvenation are biologically distinct processes (B. Zhang and Gladyshev 2020). *In vivo* pooled screening could be used to screen for combinations of perturbations that reverse age related changes while preserving cell type identity and without the formation of teratomas. Recently, such an approach has been taken *in-vitro* but could be extended to the more complex *in-vivo* setting (Roux et al., 2021). These approaches would synergize with new approaches on the perturbation side, such as improved epigenome editors (Nuñez et al., 2021), in support of combinatorial perturbations, e.g., generalizations of the famous combinations of Yamanaka factors used for induction of pluripotent stem cells. More advanced future approaches could potentially modulate the duration of expression of different such factors (Chassin et al., 2019), and barcode such timing patterns at the single cell level as well, or vary the relative intensity of expression of the factors using barcoded promoter libraries. On the readout side, single-cell lineage analysis may make it possible (Biddy et al., 2018) to relate reprogramming perturbations to the proliferative properties of cells, although this method has not yet been applied *in vivo.* Also, combinations of single-cell transcriptome sequencing with chromatin accessibility (scATAC-seq) and chromatin modification (Bartosovic et al., 2021), could be used to refine our understanding of how such perturbations impact epigenetic cell state specification.

More speculatively, it is interesting to ask whether more dynamic properties of cells can be encoded as a readout for pooled screening. At present, readouts for *in vivo* pooled screens are destructive, and longitudinal data is only available when biopsies (e.g. of liver or blood) can be acquired without sacrificing the animal. Recently, researchers have found ways to encode cell-cell interactions into a DNA readout (Clark et al., 2021), and others are studying prototype molecular recording systems (Tanna et al., 2020; Loveless et al., 2021) that could encode aspects of the history of dynamic gene expression into static readout (Rodriques et al., 2021), including with single-cell precision (Chan et al., 2019).

While previous studies have mostly focused on common laboratory mouse models, a key advantage of the pooled screening approach is its ability to obtain a large amount of information from a single animal, and thus it could be applicable to less common animal models as well. Genetically diverse mouse strains would be a simple example, but long-lived species like naked mole rats should be accessible as well, as both the viral vectors used for delivery of perturbations and the CRISPR proteins used as effectors tend to generalize well across mammalian species (Salganik et al., 2015). A pooled screen in naked mole rats could, for example, knock out putative tumor suppressors (or the whole genome) unique to this organism in the presence of tumorigenic agents, and identify actual tumor suppressors by sequencing barcodes present in tumors, and thereby help explain by what mechanisms naked mole rats are so resistant to tumors—even at high age.

Overall, by directly perturbing causal mechanisms in their intact-system context, while reading out high-dimensional signatures at a level of resolution that can reveal both shared features as well the heterogeneity of cell, cell-type and organ specific responses, *in vivo* pooled screening approaches are likely to become a general platform for accelerated study of the biology of aging, the diseases that result from it, and perturbations that seek to reverse it.

## Abbreviations

AAV, adeno-associated virus; CRISPR, clustered regularly interspaced short palindromic repeats; GFP, green fluorescent protein; LNP, lipid nanoparticle; MOI, Multiplicity of infection; RNAi, RNA interference; SASP, senescence-associated secretory phenotype; scRNAseq, Single-cell RNA sequencing; shRNA, short hairpin RNA; UMI, unique molecular identifier.
