# Corrigendum: *In Vivo* Pooled Screening: A Scalable Tool to Study the Complexity of Aging and Age-Related Disease

**DOI:** 10.3389/fragi.2021.804856

**Published:** 2021-12-03

**Authors:** Martin Borch Jensen, Adam Marblestone

**Affiliations:** ^1^ Gordian Biotechnology, San Francisco, CA, United States; ^2^ Astera Institute, San Francisco, CA, United States; ^3^ Federation of American Scientists, Washington D.C., CA, United States

**Keywords:** *in vivo*, pooled screening, direct *in vivo* screening, aging models, animal models, gene therapy, single cell sequencing, barcoding

In the original article, there was a mistake in Table 1 as published. A typo caused the entry for “Optimal payload size” for AAV to be listed as 3–4.5 kb, rather than the intended 4–4.5 kb. The corrected Table 1 appears below.

**TABLE 1:** Delivery modalities for *in vivo* pooled screening

	AAV	Lentivirus	Adenovirus	Lipid nanoparticle (with mRNA/siRNA)	siRNA/antisense oligos
Targetable tissues and cell types	Many (liver, muscle, brain, eye, lung, heart, and more)	Many	Many	Mainly hepatocytes, vasculature reported	Mainly liver and kidney, neurons with direct injection
Inter- and intra-tissue spread	Medium-high	Low	Low	Medium	High
Duration of treatment possible	Stable episomal expression in non-dividing cells for months+	Stable integration in dividing and non-dividing	Stable episomal expression in non-dividing cells for months+	Days to weeks, unless gene editing modalities delivered	Days to weeks
Optimal payload size	4–4.5 kb	7–8 kb	8–30 kb	Any	<100 bp
Payload vector construction	Moderate	Moderate	Hard	Easy	Easy but expensive
Immunogenicity	Low	Medium	High	Low	Low-High

The authors apologize for this error and state that this does not change the scientific conclusions of the article in any way. The original article has been updated.

